# Effect of three remplissage techniques on tendon coverage and shoulder kinematics: a navigated robotic biomechanical study

**DOI:** 10.1186/s12891-015-0856-z

**Published:** 2016-01-04

**Authors:** Chung Hui James Tan, Tim Saier, Constantin von Deimling, Frank Martetschläger, Philipp Minzlaff, Matthias J. Feucht, Horazio Martinez, Sepp Braun, Andreas B. Imhoff, Rainer Burgkart

**Affiliations:** Department for Orthopedic Sports Medicine, Technische Universität München, Munich, Germany; Department of Orthopaedics -Biomechanics Laboratory, Technische Universität München, Ismaninger Straße 22, Munich, 81675 Germany; Berufsgenossenschaftliche Unfallklinik Murnau, Murnau, Germany; Chirurgische Klinik Dr. Rinecker, Munich, Germany; Department of Orthopedic Surgery and Traumatology, Freiburg University Hospital, Freiburg, Germany

**Keywords:** Shoulder dislocation/instability, Hill-Sachs defect, Remplissage, Tendon coverage, Torque, Kinematics

## Abstract

**Background:**

In addition to Bankart repair engaging Hill-Sachs defects in glenohumeral instability have been treated successfully with remplissage procedure. The purpose of this study was to compare three remplissage techniques regarding (I) ability of preventing Hill-Sachs defect from engaging, (II) influence on glenohumeral rotational torque, and (III) resulting tendon coverage over the Hill-Sachs defect.

**Methods:**

Standardized engaging Hill-Sachs defects and Bankart lesions were created in *n* = 7 fresh frozen human shoulder specimens. Besides Bankart repair three remplissage techniques (T) with double anchor position in the valley of the defect zone were studied: T1, knots tied over anchors; T2, knots tied between anchors (double-pulley); T3, knotless anchors with a suture tape.

A parallel position-orientation and force-moment controlled navigated roboticsystem was used to compare prevention of Hill-Sachs defect engagement and torque [Nm]. Pressure sensitive film was used to study area of infraspinatus tendon coverage over Hill-Sachs defect [%].

**Results:**

All remplissage techniques prevented engagement of the Hill-Sachs defect without showing any construct failures. Regarding humeral torque there were significant impairments observed between intact conditions and the three investigated repair techniques in 60° abduction and ≥30° external rotation (*p* < .04). There was no significant difference in torque between intervention groups (n.s.). With a mean coverage of 26.8 % over the defect zone the knotless suture tape technique (T3) significantly improved area of soft tissue coverage compared to the other techniques (*p* = .03).

**Conclusion:**

All remplissage techniques prevented engagement of the Hill Sachs defect. With high abduction and external rotation ≥30° all techniques showed significant higher humeral torque compared to the intact specimens, while there was not one technique superior over the others. The suture tape technique conferred the largest and most effective area of tendon coverage over the Hill-Sachs defect zone. Long-term success of the remplissage procedure can possibly be enhanced by increasing the interface area of tendon coverage over the Hill-Sachs defect. Clinical studies will be necessary to proof potential benefits for clinical outcome.

## Background

Engaging Hill-Sachs defects of the humeral head are well documented risk factors for recurrent glenohumeral shoulder instability and failed anterior shoulder stabilization [1–3]. Several studies have shown that an engaging Hill-Sachs defect treated with Bankart repair alone is associated with a high re-dislocation rate [1, 3–5]. Different surgical options have been described in order to address this problem: Retrograde disimpaction, osteochondral allograft, humeral osteotomy and partial or complete replacement of the humeral head [6–9].

Connolly first described an open procedure of filling a Hill-Sachs defect with infraspinatus and capsule tenodesis, together with a Bankart repair [10]. This technique works by converting an intra-articular defect into an extra-articular one and thus preventing the defect from engaging at the anterior glenoid rim. Furthermore, there is a check-rein effect on the humeral head, effected by the fixed infraspinatus tendon and posterior capsule. Wolf et al. termed the procedure remplissage when they performed the procedure arthroscopically, placing suture anchors into the valley of the Hill-Sachs defect and passing the sutures posteriorly through the infraspinatus tendon and capsule [11]. Cadaveric studies have shown that medium sized Hill-Sachs lesions without glenoid bone loss can be successfully treated with the remplissage procedure if combined with Bankart repair [12, 13]. In recent years, clinical studies have already reported good functional outcomes with minimal loss of shoulder range of motion [14–16]. Although, filling of the Hill-Sachs defect by infraspinatus tendon and capsule on post-operative scans has been demonstrated [17], there are no studies in the literature investigating the amount of infraspinatus tendon coverage over the defect zone produced by different techniques.

The aim of this in-vitro biomechanical study is to compare three arthroscopic remplissage techniques, each with suture anchors placed in the valley of a standardized Hill-Sachs defect, but with varying methods of fixation of suture materials. The study uses a novel parallel position-orientation and force-moment controlled navigated robotic model. Robotic parallel control was used to minimize forces in the orthogonal axis [18, 19]. At the same time, this allowed for external rotations while maintaining a constant forward flexion and abduction angle with centered humeral head within the glenoid. The effect of each remplissage technique on (I) glenohumeral engagement, (II) glenohumeral torque (Nm), and (III) area of tendon coverage over the Hill-Sachs defect zone (%) was quantified.

It was hypothesized that a knotless suture tape technique confers a larger area of tendon coverage over the defect zone compared to suture tying above the anchors and/or tying between the anchors (double-pulley) with no differences in defect engagement and glenohumeral torque.

## Methods

This study was approved by the Ethics Committee of the Faculty of Medicine of the Munich University of Technology (ID number: 339/14).

### Specimen preparation

Seven fresh-frozen human cadaveric shoulder specimens (4 right/3 left, 7 male, mean age 46.8 years) were thawed and dissected, removing all soft tissue below the deltoid tuberosity of the humerus and the inferior half of the scapula. Shoulders with macroscopic signs of osteoarthritis or other pathologic joint conditions were excluded from this study. The scapulae (in 10° forward inclination) and humeri (transected at 7 cm distal to the surgical neck) were then embedded in a polyurethane resin (RenCast® FC 53 Isocyanate / FC 53 Polyol, Huntsman, Belgium). Passive marker tracking tools were rigidly attached to the scapulae and humeri in order to record the position and orientation data during the testing procedure.

### Preparation of Bankart lesion and Hill-Sachs defect

Since several studies described Hill-Sachs defects ≥25 % to be relevant for recurrent glenohumeral instability a 30 % defect size was chosen [2, 20]. The standardized Hill-Sachs defects were created using the method described by Sekiya et al. [20] An extended horizontal capsulotomy was performed to expose the antero-inferior rim of the glenoid and the posterior aspect of the humeral head. A line was drawn on the humeral head parallel to the anterior-inferior glenoid rim, representing the defect orientation. The humeral head diameter was measured with a caliper [mm]. A defect equivalent to 30 % of the postero-lateral humeral head was marked on the humeral head and created with an oscillating saw. The positions of suture anchors were marked in the valley of the defect, at the junctions of one-thirds of the defect length. The defect size, shape and suture anchor location were then duplicated on a template. Bankart lesions were created by sharp dissection of the labrum from the anterior-inferior glenoid rim. After defect creation the capsulotomy was anatomically closed without any over-tightening and/or overlap. Thus careful attention was paid to maintain the initial capsular tension. Finally subscapularis and teres minor muscles were tagged together.

### Preparation of pressure-sensitive film

Using pressure-sensitive films (Prescale, Super Low Pressure Fuji Photo Film, Fuji Photo Film Co Ltd, Tokyo, Japan; pressure sensitivity range: 0.5 to 2.5 MPa), contact area between musculus infraspinatus tendon and Hill-Sachs defect was determined [%]. By use of individually prepared templates the films were cut to match the standardized Hill-Sachs defects. They were sealed and waterproofed within thin polyethylene sheets to be fixed on the bony surface of the Hill-Sachs defect using an adhesive agent. By doing so, correct positioning of the film was ensured throughout testing. Immediately after testing, the films were removed and digital images were created with a color scanner.

### Surgical techniques

#### Bankart repair

Bankart repair was completed by placing 2 suture anchors (3.5 mm, Arthrex Inc., Naples, USA) at 4:30- and 6-o’clock positions on the glenoid and passing the sutures through the labrum using a horizontal mattress technique.

#### Remplissage

The distance between the suture anchors in the Hill-Sachs defect was measured [mm]. Two sites were marked on the infraspinatus tendon overlying the valley of the defect, with their location corresponding to the distance between the suture anchors. Sutures were passed through the same sites, ensuring that equal amount of tendon tissue was compressed.

**Fig. 1:**
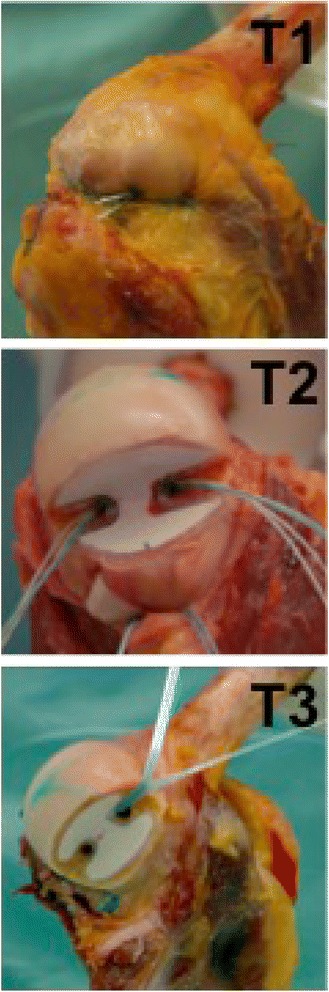
Three arthroscopic remplissage techniques (T): T1 knot technique, T2 double-pulley technique, T3 knotless suture tape technique

Three remplissage techniques were used (see Fig. 1):

- *Technique 1* (T1), as originally described by Purchase et al., was performed by inserting 2 double-loaded 5.5-mm anchors (Biocorkscrew, Arthrex Inc., Naples, USA) into the valley of the Hill-Sachs defect and passing horizontal mattress sutures through the infraspinatus tendon with surgical knots over the anchors [21].
- *Technique 2* (T2) involved placing of the anchors and passing of the sutures as in T1; however, sutures were tied using the double pulley technique described by Koo and Burkhart [22]. For T1 and T2, the pressure sensitive film was placed after the insertion of both anchors and passage of sutures through the infraspinatus tendon. Sutures were then tied to effect compression of the infraspinatus tendon and posterior capsule.
- *Technique 3* (T3) involved placement of two 4.75 mm knotless anchors (Swivel Lock, Arthrex Inc., Naples, USA) in the valley of the Hill-Sachs defect. The first knotless anchor was inserted with one end of a suture tape (Fibertape, Arthrex Inc., Naples, USA) and the tape was passed through the infraspinatus. The next knotless anchor was then inserted together with the tape through the infraspinatus tendon and into the valley of the Hill-Sachs defect. After pre-tensioning of the tape, by inserting the anchor according to the manufacturers recommendation, the tape was evenly tensioned. For T3 the pressure sensitive film was adhered to the defect before placement of the second knotless anchor. Care was taken to ensure that the second knotless anchor did not touch the pressure sensitive film. Subsequently the inferior capsule was repaired with sutures.

#### Testing apparatus

**Fig. 2:**
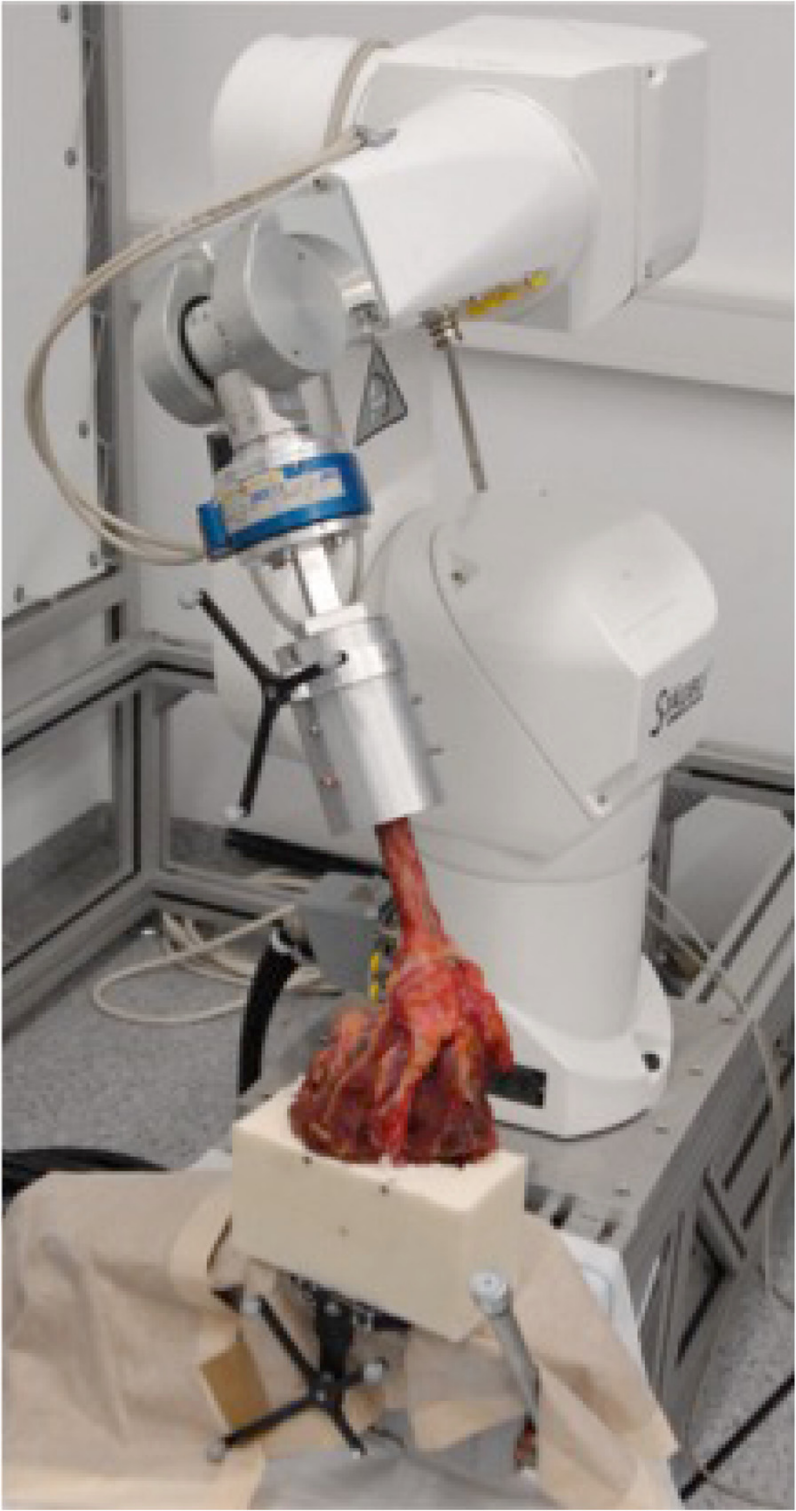
Shoulder specimen with the robotic testing system. Legend Table 1: T1: knot technique, T2: pulley technique, T3: tape technique, I: Intact native specimens. Legend Table 2: T1: knot technique, T2: pulley technique, T3: knotless suture tape technique (T3 vs. T1/T2 *p* = .03; T1 vs. T2 n.s.)

An industrial robot (Stäubli RX 90-B, Pfäffikon, Switzerland) was used to perform a defined motion of the humerus, while the scapula was in a fixed position (Fig. 2). A force torque sensor (FTS) (6 DoF JR3 Inc., Woodland, CA, USA) with a resolution of 0.1 N and 0.005 Nm was chosen to collect the force-moment data. A coordinate system associated with the scapula was used to define motion of the humerus with respect to the scapula, as previously described by Sekiya et al. implementing some minor modifications: The x-axis was defined as being perpendicular to the scapular plane and directed anteriorly. Rotation about the x-axis described abduction in the scapular plane [20]. The z-axis lies along the longitudinal axis of the humerus and rotation about the z-axis axis described internal-external rotation. The y-axis was defined as a cross product of the two already defined axes and following the right hand rule.

An optical tracking system (Polaris, Northern Digital Inc., Waterloo, Canada) was used to measure positions and movements of the humerus in respect to the scapula throughout testing.

To the knowledge of the authors, this navigated and force-moment controlled robotic setup represents a novel model for biomechanical evaluation of glenohumeral stability and range-of-motion.

#### Testing protocol

Reference trajectories for external rotation of the humerus were performed by hand and recorded by the optical tracking system. These data were converted into the coordinate system of the robot and used as position reference trajectory. Robotic parallel control was used in order to apply a humeral compressive force of 22 N to the scapula, minimizing forces in the orthogonal axis [18, 19]. At the same time, this allowed to perform external rotations maintaining a constant forward flexion and abduction angle and centered the humeral head within the glenoid. Furthermore, this method ensured that the humerus rotated about a biological and not a mathematical axis. The parallel control parameters were adjusted in a pre-test that the force control action (compressive force of 22 N) prevails over the position control action (external rotation replay of the reference trajectory).

The study protocol was designed to examine effects of rotational glenohumeral torque [Nm] with glenohumeral abduction angles of 0°, 30°, and 60° and external rotation angles of 0°, 30°, and 60°. Forward flexion was kept constant at 0° throughout all experiments. These positions were chosen, because of the final positions clinical relevance [2]. Most commonly antero-inferior glenohumeral instability is associated with a combined 90° abduction and 90° external rotation of the shoulder. Under laboratory conditions this was realized with 60° of glenohumeral abduction and 60° of glenohumeral external rotation of the specimens, given that the scapulothoracic joint accounts for another 30° of abduction and external rotation *in vivo* [2]. Therefore the final laboratory glenohumeral position corresponds to the above mentioned clinical relevant shoulder position.

Each specimen was preconditioned at 0° of abduction with 3 cycles of maximum external rotation. Measurements of the shoulder rotated through the arc of external rotation (≤60°) were taken at 0°, 30°, and 60° of abduction. The force sensor attached to the robot-arm recorded force-moment signals.

#### Glenohumeral engagement and dislocation testing

Prior to performing Bankart repairs and remplissage procedures a glenohumeral dislocation test was performed to confirm engagement and consecutive antero-inferior dislocation in all specimens. For this reason with 60° of glenohumeral abduction and external rotation a centralizing force of 22 N and an anterior-inferior directed 30 N force was applied by the robot. Glenohumeral engagement and dislocation was analyzed visually and by force profile change.

The same test was repeated after Bankart repairs and remplissage procedures have been performed. Glenohumeral integrity was quantified as the absence of anterior dislocation with engagement detected visually and by comparison to the force profile of the intact specimens.

#### Data collection pressure contribution

Digital scans of the pressure sensitive films were measured using Image-Pro Plus software (Media Cybernetics Inc., Rockville, MD, USA) and expressed in percentage of total film area vs. tendon coverage over defect area.

#### Statistical analysis

A power analysis was performed a priori. The analyses indicated that *n* = 7 specimens were required to ensure at least . Nine power for all outcome variables.

Statistical analysis was performed using SPSS software (version 13.0, SPSS, Chicago, Illinois). The level of statistical significance was defined as *p* < 0.05.

Data on ratio of area of tendon compression to the total area of the film is expressed as percentage value. Data for range of motion is represented my mean, standard deviation (SD), and 95 % confidence interval (95 % c.i.). Statistical analysis for differences between the groups was determined by one-way ANOVA [23]. Post-hoc test for differences of dependent variables was conducted using Tukey HSD.

## Results

### Prevention of glenohumeral engagement

Prior to Bankart repair and remplissage stabilization all specimens (7/7) showed engagement and dislocated during dislocation testing. After stabilization, all intervention groups (T1, T2, and T3) showed no engagement and/or antero-inferior glenohumeral dislocation in 7 out of 7 specimens.

### Glenohumeral torque

Data on glenohumeral torque in 0°, 30°, and 60° of glenohumeral abduction and 0°, 30°, and 60° external rotation is summarized in Table 1.

**Table 1:** External rotational glenohumeral torque [Nm] of three remplissage techniques compared to intact native specimens

0° gh-Abduction		Mean [Nm]	SD	95 % CI for Mean		*p*-value
				Lower Bound	Upper Bound	
0° ER	Intact	0	0.01	0	0.01	
	Knot (T1)	0	0.04	−0.03	0.04	n.s.
	Pully (T2)	−0.01	0.03	−0.04	0.02	n.s.
	Tape (T3)	0	0.01	−0.01	0.02	n.s.
30° ER	Intact	0.04	0.06	−0.02	0.1	
	Knot (T1)	0.09	0.13	−0.02	0.21	n.s.
	Pully (T2)	0.17	0.18	0.01	0.34	n.s.
	Tape (T3)	0.12	0.13	0	0.24	n.s.
60° ER	Intact	0.04	0.14	−0.09	0.17	
	Knot (T1)	0.2	0.33	−0.1	0.5	n.s.
	Pully (T2)	0.26	0.25	0.03	0.5	n.s.
	Tape (T3)	0.21	0.33	−0.1	0.52	n.s.
30° gh-Abduction						
0° ER	Intact	0	0.01	−0.01	0.01	
	Knot (T1)	−0.02	0.02	−0.04	0.01	n.s.
	Pully (T2)	−0.02	0.04	−0.06	0.01	n.s.
	Tape (T3)	−0.01	0.02	−0.03	0.01	n.s.
30° ER	Intact	0.05	0.06	0	0.11	
	Knot (T1)	0.08	0.06	0.02	0.14	n.s.
	Pully (T2)	0.15	0.14	0.02	0.28	n.s.
	Tape (T3)	0.08	0.07	0.02	0.14	n.s.
60° ER	Intact	0.06	0.11	−0.05	0.17	
	Knot (T1)	0.1	0.13	−0.02	0.23	n.s.
	Pully (T2)	0.17	0.14	0.04	0.3	n.s.
	Tape (T3)	0.09	0.09	0.01	0.17	n.s.
60° gh-Abduction						
0° ER	Intact	0	0.01	−0.02	0.01	
	Knot (T1)	0	0.02	−0.01	0.02	n.s.
	Pully (T2)	0	0.02	−0.02	0.02	n.s.
	Tape (T3)	0.01	0.02	−0.01	0.02	n.s.
30° ER	Intact	0.05	0.06	0	0.11	
	Knot (T1)	0.13	0.08	0.06	0.2	0.05
	Pully (T2)	0.14	0.07	0.08	0.2	0.02
	Tape (T3)	0.14	0.1	0.05	0.23	0.02
60° ER	Intact	0.05	0.09	−0.03	0.13	
	Knot (T1)	0.15	0.11	0.05	0.25	0.02
	Pully (T2)	0.18	0.1	0.08	0.28	0.04
	Tape (T3)	0.15	0.07	0.08	0.21	0.00

All remplissage techniques showed significant higher torque with 60° abduction and ≥30° external rotation (*p* < .04). There were no differences observed between the three techniques (n.s.).

### Area of M. infraspinatus tendon coverage over Hill-Sachs defect

Semi-quantitative analysis of defect coverage patterns showed increased compression around and between the insertion points of each anchor and the edges of the Hill-Sachs defect for all repair groups (T1, T2, and T3). The main area of compression was located in the center of the bony defect for any repair group.

The mean coverage of M. infraspinatus tendon into the Hill-Sachs defect was largest for the tape technique (T3) with 26.8 %. The pulley technique (T2) revealed a 15.9 % coverage, and the knot technique (T1) a 13.3 % coverage. Area of tendon coverage over defect zone was in T3 significantly larger than in T1 and T2 (*p* = .03). There was no significant difference between T1 and T2 (n.s.). See Table 2.

**Table 2:** Area of M. infraspinatus tendon coverage [%] over standardized Hill-Sachs defect

	Technique	Area	*p*-value
Percentage of M. infraspinatus tendon coverage over total Hill-Sachs defect zone	T1 (Knot)	13.3 %	
	T2 (Pulley)	15.9 %	
	T3 (Tape)	26.8 %	0.03

## Discussion

The most important finding of this biomechanical study is that the proposed knotless suture tape technique provides significant superior coverage of the infraspinatus tendon over the Hill-Sachs defect zone. All three techniques revealed stable joints without engagement. There was no difference in humeral torque between groups. Therefore, the studies hypothesis was proofed. Hill-Sachs remplissage is a non-anatomic arthroscopic soft tissue procedure performed in order to add extra stability to a conventional Bankart repair. Several clinical studies have already shown good and reliable clinical results for this “add-on” technique. [14–16, 24–26] However, some reports have suggested, that patients undergoing a remplissage procedure might suffer from postoperative glenohumeral rotational deficits [14, 27]. In contrast, a recent systematic review indicated no significant loss of shoulder motion following the procedure [28].

Several different techniques to perform a remplissage procedure have been described in literature and a gold standard has not been defined yet. In order to better understand the effect of different remplissage procedures on joint kinematics and stability, a few biomechanical studies have been conducted [12, 29–32].

A study by Elkinson et al. also investigated the effects of 3 different remplissage techniques on shoulder kinematics. They found that medial suture passage through the posterior soft tissue consistently led to the greatest mean restriction of range of motion and the highest stiffness values [12].

The results of the presented study show that all three investigated remplissage techniques re-stabilized the glenohumeral joint and prevented engagement of the defect sufficiently. Regarding influence on glenohumeral torque, all three intervention groups showed significantly impaired torque with 60° abduction and ≥30° external rotation compared to anatomical conditions. There was no difference observed between techniques. This in vitro finding supports current clinical, as well as biomechanical findings [12, 14, 15, 22].

Regarding area of tendon coverage over the Hill-Sachs defect, this study showed that the proposed knotless suture tape technique (T3) created a significantly larger area of tendon coverage over the defect zone compared to the two other techniques (T1, T2). The authors are not aware of any literature investigating and reporting on area of tendon coverage over the defect zone after remplissage.

For rotator cuff repair biomechanical and clinical studies have shown the critical influence of tendon coverage over the footprint for successful healing [33, 34]. In the opinion of the authors, in addition to the initial tenodesis or check-rein effect, optimal healing of posterior tissues into the defect is important for remplissage as well. Thus it is logical that anchors be placed in the valley of the defect for healing to occur. In consequence the authors propose a new technique, using knotless anchors and suture tape, aiming to increase interface of tendon coverage and yet have not more limitation of glenohumeral torque compared to other techniques.

This study has inherent weaknesses, including the limitation of applying a biomechanical model to a clinical problem. Using a simplified cadaveric model, important factors contributing to shoulder stability such as dynamic muscle activation were not considered. Nevertheless, with dynamic range of motion the joint compression force was incorporated in this in vitro model [20, 35]. Furthermore, the results from this time zero study do not account for any phenomenon of biological healing e.g. formation of scar tissue, which occurs in the clinical setting and might result in decreased ROM. Postoperative soft tissue stretching, which may minimize the restrictive effects of the remplissage over time could also not be investigated with this study. However, the setup has shown to be reproducible, reliable and capable of answering the stated hypothesis.

The strengths of the study include the use of an industrial robot and an optical measurement system, providing a high accuracy for joint stability and kinematics testing. Repairs only differed by repair technique and were all performed by a single fellowship trained orthopedic surgeon. Finally, the native specimens were used for comparison mimicking a “pre- vs. postoperative” change of shoulder kinematics.

## Conclusions

All remplissage techniques prevented engagement of the Hill Sachs defect without luxation. With high abduction and external rotation ≥30° all techniques showed significant higher humeral torque compared to the intact specimens, while there was not one technique superior over the others. The suture tape technique conferred the largest and most effective area of tendon coverage over the Hill-Sachs defect zone. In the clinical setting, the increased tendon coverage might lead to better healing at the soft tissue—bone interface. Clinical studies will be necessary to proof potential benefits for outcome.
